# Hemophagocytic Lymphohistiocytosis (HLH) in a Patient with Disseminated Histoplasmosis

**DOI:** 10.1155/2020/5638262

**Published:** 2020-07-18

**Authors:** Neeraja Swaminathan, Jorge M. Vinicius, Jesse Serrins

**Affiliations:** Albert Einstein Medical Center, Philadelphia, PA 19141, USA

## Abstract

Hemophagocytic lymphohistiocytosis (HLH) is a rare condition characterized by an overwhelming inflammatory cascade activation which is often associated with rapid progression and high mortality. It may be familial with an underlying genetic mutation or triggered by infection, malignancy, and autoimmune disease. Disseminated histoplasmosis caused by histoplasma capsulatum is a granulomatous fungal disease seen typically in immunocompromised patients with varied clinical manifestations and requires long-term antifungal therapy. We present the case of a 61-year-old immunocompromised female with significant travel history who came with fever, pancytopenia, and liver failure raising suspicion for HLH that prompted a bone marrow biopsy procedure. Hemophagocytic figures consistent with HLH and numerous encapsulated fungi resembling histoplasma were visualized. She was treated with intravenous (IV) liposomal amphotericin B. Etoposide chemotherapy and interleukin-1 (IL-1) antagonist anakinra were deferred in order to limit her immunosuppression, and treatment was focused on antifungal therapy.

## 1. Introduction

Hemophagocytic lymphohistiocytosis is a life-threatening condition associated with an inflammatory cytokine surge leading to multiorgan failure. Acquired HLH is described typically in adult patients who have a clear trigger such as infection, malignancy, and autoimmune conditions. This serious condition needs prompt recognition and management; otherwise, mortality is extremely high. Diagnosis of HLH requires 5 out of the following criteria: fever, splenomegaly, cytopenia affecting >2 lineages, hypertriglyceridemia and/or hypofibrinogenemia, hemophagocytosis in the marrow/spleen/lymph node or liver, low or absent natural killer (NK) cell activity, high ferritin, and elevated soluble CD25 [1, 2]. Normally NK cells play a central role in the response to intracellular infections. In HLH, there is a defect in NK cell response which leads to a positive feedback loop of uncontrolled intracellular infection and ongoing immune activation [2, 3].

Histoplasmosis is an endemic mycosis that is common in many tropical countries, South America, and also in the Midwestern states of the US. Initial exposure is usually by inhalation of the conidia into the respiratory tract, and dissemination occurs usually due to reactivation of histoplasmosis secondary to immunosuppressive status. Diagnosis by urine antigen is the most sensitive (90% sensitivity) and rapid method. However, the gold standard is a fungal culture which may take several weeks to result. Lymph node/bone marrow biopsy to demonstrate the histoplasma yeast form is also useful to make a quick diagnosis [4–6]. Disseminated histoplasmosis has certainly been described in the past to be a trigger for HLH. However, in the setting of an underlying infection, the management approach for HLH has not been well defined. Disseminated histoplasmosis is a fulminant infection which needs long-term systemic antifungal therapy. Immunosuppressive therapy can potentially worsen the underlying infection especially since histoplasmosis is typically seen to affect immunosuppressed individuals such as those with HIV or taking chemotherapy [3–5]. In this case report, we would like to discuss diagnosis and management of HLH and the spectrum of presentation of disseminated histoplasmosis and its treatment.

## 2. Case Presentation

A 61-year-old female presented with past medical history of rheumatoid arthritis (RA) on long-term treatment with methotrexate and infliximab came with fevers, upper respiratory symptoms, fatigue, and myalgia for 1 week which notably began a few weeks after she returned from travel to Brazil and Panama. She was empirically managed with oseltamivir for suspected influenza; however, she had no improvement in her symptoms. She was admitted at a local hospital where she was noted to have significantly elevated liver enzymes along with thrombocytopenia, elevated international normalized ratio (INR), and creatinine. She was placed on broad-spectrum antibiotic coverage (piperacillin-tazobactam, vancomycin, doxycycline, and micafungin) at the outside hospital. In view of clinical deterioration, the patient was transferred. At presentation, she was noted to be alert and oriented, tachycardic, tachypneic, and normotensive. Chest radiography showed diffuse bilateral pulmonary infiltrates which was concerning for fluid versus multifocal pneumonia. She was pancytopenic, had elevated ferritin level, and her peripheral smear showed toxic granulations within the granulocytes, rare inclusions, and Dohle bodies.

Differentials considered for this patient who was a recent traveler, immunocompromised (due to RA treatment) presenting with pyrexia of unknown origin, pancytopenia, acute liver failure, and respiratory distress, included acute viral hepatitis (A, B, C, and E), cytomegalovirus (CMV), Epstein–Barr virus (EBV), etc. Apart from infections, possibility of malignancy or autoimmune etiology given the history of rheumatoid arthritis was also considered. She had computed tomography (CT) of the chest and abdomen which showed hepatomegaly, bilateral pleural effusions, extensive ground glassing, and septal thickening. In view of such a broad differential in a critically ill patient, she was continued on broad antibiotic coverage as noted above. Since she met 5 out of 8 criteria for HLH including peripheral smear findings concerning for possible hemophagocytosis, she was empirically initiated on IVIG 0.4 g/kg and dexamethasone 10 mg/m^2^.

**Figure 1:**
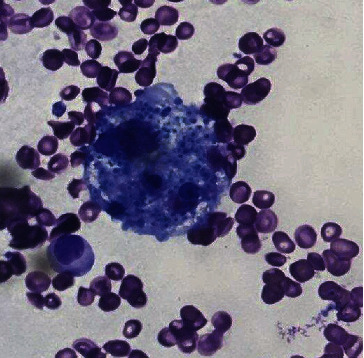
Hemophagocytosis on bone marrow smear (100x magnification).

**Figure 2:**
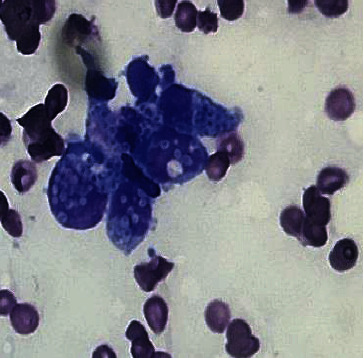
Encapsulated fungus in the cytoplasm of mononuclear cells on bone marrow smear (100x magnification).

**Figure 3:**
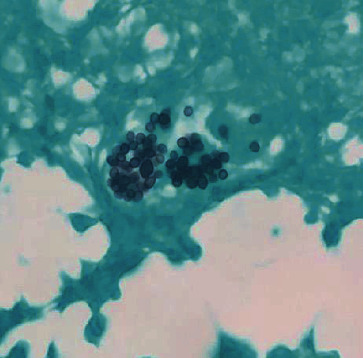
Budding fungus from intracellular to extracellular space on silver staining of mononuclear cells on bone marrow smear (100x magnification).

She had active bleeding from her IV-line site secondary to coagulopathy which was managed with blood product transfusions. Her respiratory status continued to worsen despite noninvasive ventilation, and she had significant hypoxia as a result of which she was intubated on day 4 of her admission. After she was clinically stabilized, she underwent a bone marrow biopsy. The aspirate smear was positive for hemophagocytosis (Figure 1). Additionally, it also showed some inclusions on the marrow that were highly suspicious for a fungal infection (Figure 2). Encapsulated organisms with narrow based budding positive on silver stain were confirmed which clinched the diagnosis of disseminated histoplasmosis (Figure 3). Urine histoplasma antigen was sent which was >25 U/mL. Giemsa staining, immunohistochemistry, and polymerase chain reaction (PCR) for leishmania were all negative. Cryptococcal antigen and HIV testing were also negative. Fungal cultures—blood and sputum—were sent, and the patient was started on IV liposomal amphotericin B (5 mg/kg) and all the other broad-spectrum empiric antibiotics were stopped.

Since the patient had HLH secondary to disseminated histoplasmosis, it was decided to limit immunosuppression, and therefore steroids were also discontinued. After initiation of amphotericin, the patient had steady and gradual clinical improvement along with improvement in her liver function tests and her leukocyte count (see Table 1).

Subsequently, the patient developed thrombocytopenia and petechial rash on her back and had increasing creatinine. In view of this, she was switched to itraconazole after 9 days of treatment with amphotericin. However, after discontinuation of amphotericin, there was further clinical deterioration and the patient had worsening shock and new fever spikes and developed gastrointestinal bleeding and disseminated intravascular coagulation (DIC). She was supported with blood products and was also started on vancomycin and cefepime in view of concern for new nosocomial infection. Since inadequate amphotericin treatment was also considered, it was resumed on day 17 of admission. Unfortunately, despite her initial hematological response and aggressive treatment measures, she had refractory shock and multiorgan failure and eventually succumbed on day 19 of her admission.

## 3. Discussion

Histoplasma capsulatum is a ubiquitous dimorphic fungus and usually presents as a pulmonary infection. However, in immunocompromised hosts such as HIV infected individuals, there can be systemic spread. Treatment with tumor necrosis factor (TNF) inhibitors such as infliximab can also lead to increased risk of opportunistic infections particularly granulomatous diseases such as histoplasmosis and tuberculosis. Symptoms of disseminated histoplasmosis usually include fever, fatigue, weight loss, hepatosplenomegaly, lymphadenopathy, skin lesions, and pancytopenia [4, 6–8]. There is considerable overlap of these symptoms with primary HLH, and the infection can itself trigger HLH. HLH simulates a variety of conditions such as sepsis, acute viral hepatitis, lymphoproliferative disorders, and thrombotic thrombocytopenic purpura, making it difficult to recognize. It is characterized by a hyperstimulated but ineffective immune response especially in the NK cells. It is because of this that intracellular pathogens such as EBV, CMV, coxiella, mycobacteria, and fungi are more likely to trigger HLH [9]. HLH is characterized by low/absent NK cell activity, defective cytotoxic cell function, and unopposed macrophage activity. These activated macrophages secrete ferritin. They also secrete soluble interleukin-2 (IL-2) receptor which is also referred to as CD 25. Elevated levels of ferritin and soluble CD25 can be used to distinguish it from other mimics [2, 3].

In this patient, prior to establishing histoplasmosis, there were other potential triggers to be considered for HLH such as her underlying rheumatoid arthritis or a malignancy. Although the patient had well controlled symptoms of her RA, her exact premorbid immunological status was not known since records of her CD4 and CD8 counts or immunoglobulin levels were unavailable. Hence, this patient received a 5-day course of IVIG and steroids. This was stopped after confirmation of the infection. Initiation of chemotherapy with etoposide and IL-1 antagonist therapy with anakinra for treatment of HLH were also considered but deferred because the patient had notable improvement clinically and in her laboratory parameters after initiation of amphotericin.

Guidelines from the Infectious Disease Society of America (IDSA) state that disseminated histoplasmosis requires at least 1-2 weeks of treatment with liposomal amphotericin B followed by maintenance itraconazole for 12 months [10]. After initial improvement, this patient developed rash, kidney injury, and thrombocytopenia which were attributed to amphotericin-related toxicity and the patient was switched to itraconazole after 9 days of amphotericin. However the patient continued to deteriorate and had DIC and overt gastrointestinal bleeding. It was unclear if this rapid decline was due to gastrointestinal histoplasmosis after premature discontinuation of amphotericin, progression of amphotericin treatment toxicity, or perhaps sepsis secondary to a new nosocomial infection acquired in the intensive care unit [11].

This patient received empiric antifungal treatment with micafungin. In hindsight, amphotericin B may have been a more prudent choice given its robust antifungal activity against histoplasma. However, this benefit needs to be weighed against the risks of its adverse effects including infusion reaction and nephrotoxicity. Ultimately, the choice of antifungal depends on which kind of mycosis is being suspected. In this immunocompromised patient who was also a returning traveler, there were several mycoses that were on the differential and it was challenging to prioritize one over the other.

There is limited data regarding the role of immunosuppressive therapy and IVIG in the setting of an underlying infection. In a prior review of 11 patients with histoplasma-associated HLH, of the 5 patients that received immunosuppression (including steroids, IVIG), 4 died. 2 out of the 5 who got no additional immunosuppression also died. Although there were more survivors in the group that did not get immunosuppression, the authors note that the analysis was under powered [3]. In another review of 60 patients with histoplasma-associated HLH, 9 out of the 19 that died (47%) and 12 out of the 41 (29%) survivors had received etoposide or steroids/IVIG [12].

The HLH-2004 guidelines recommend treatment with etoposide, dexamethasone, cyclosporine A, and also intrathecal methotrexate as steroids in selected patients [1, 13]. However, these protocols largely pertain to primary HLH and are adapted from the pediatric population. At present, there is no consensus regarding continuation of chronic immunosuppression and also about initiating steroids or IVIG in those with an identified infectious trigger for HLH.

Thus, several pertinent clinical questions and challenges arose during the management of this patient such as role of immunosuppressive therapy in histoplasma-related HLH, optimal duration of amphotericin, and management of its toxicity.

## 4. Conclusion

This case report highlights the importance of being aware of HLH as a diagnosis in patients with fever of unknown origin especially when associated with pancytopenia and liver failure. Early recognition of a patient that meets diagnostic criteria for HLH and subsequently identifying a trigger and appropriately managing the same is vital in ensuring improved mortality outcomes in this otherwise fatal condition. Further studies are required to develop a robust treatment protocol for adult secondary HLH especially with regard to immunosuppressive therapy and IVIG in the setting of concomitant infection.

## Figures and Tables

**Table 1:** Patient's trend of laboratory values during course of admission.

Day	1	2	3	4	5	6	7	8	9	10	11	12	13	14	15	16	17	18	19
ALP (IU/L)	227	241	247	326	349	405	503	557	475	475	584	877	484	470	444	355	233	197	143
Total bilirubin (mg/dL)	7.5	9.4	10.2	9.9	8.9	9.3	8.7	8.4	6.8	5.9	5.7	7.1	5.8	5.2	5.6	4.4	4.3	4.2	3.7
Direct bilirubin (mg/dL)	6.0	7.0	8.0	7.1	6.6	6.4	6.1	6.2	5.2	4.7	4.6	5.5	4.3	2.9	4.1	3.2	3.1	3.2	2.8
ALT (IU/L)	272	340	466	405	366	428	446	400	328	242	247	246	185	172	142	105	75	55	39
AST (IU/L)	712	965	1827	1379	1319	1935	1718	1288	818	416	358	284	151	119	87	64	43	37	27
Ferritin (ng/mL)	>40,000	>40,000	>40,000	>40,000	>40,000	>40,000	>40,000	>40,000	>40,000	35548	16993	11886	8775	6157	4609	2990	1918	1521	1425
WBC (×10^3^/mcL)	2.73	2.91	2.97	1.70	1.41	2.62	5.25	9.67	9.64	9.96	10.73	11.81	11.37	10.66	9.87	9.29	7.52	9.44	9.79
Absolute lymphocyte count (cells/mm^3^)	410							1793		1827		2350		1978		1858		1888	
Hemoglobin 0 (g/dL)	7.8	6.9	7.2	6.8	7.1	7.1	8.4	7.5	7.7	6.9	7.9	6.4	7.7	7.4	7.3	7.2	6.0	5.0	3.8
Platelet (×10^3^/mcL)	40	53	57	85	91	105	110	125	128	113	72	75	57	36	44	55	58	42	30
Fibrinogen (mg/dL)	78	60	90	120	206	353	293	292	296	294	220	201	217	181	172	183	273	177	91
INR	2.5	2.4	1.7	1.4	1.5	1.2	1.2	1.2	1.1	1.1	1.3	1.1	1.1	1.2	1.2	1.2	1.5	1.9	2

ALP, alkaline phosphatase; ALT, alanine transaminase; AST, aspartate transaminase; WBC, white blood cell.
